# Threatened Bird Valuation in Australia

**DOI:** 10.1371/journal.pone.0100411

**Published:** 2014-06-23

**Authors:** Kerstin K. Zander, Gillian B. Ainsworth, Jürgen Meyerhoff, Stephen T. Garnett

**Affiliations:** 1 The Northern Institute, Charles Darwin University, Darwin NT, Australia; 2 Research Institute for the Environment and Livelihoods, Charles Darwin University, Darwin NT, Australia; 3 Institute for Landscape Architecture and Environmental Planning, Technische Universität Berlin, Berlin, Germany; University of Kent, United Kingdom

## Abstract

Threatened species programs need a social license to justify public funding. A contingent valuation survey of a broadly representative sample of the Australian public found that almost two thirds (63%) supported funding of threatened bird conservation. These included 45% of a sample of 645 respondents willing to pay into a fund for threatened bird conservation, 3% who already supported bird conservation in another form, and 15% who could not afford to pay into a conservation fund but who nevertheless thought that humans have a moral obligation to protect threatened birds. Only 6% explicitly opposed such payments. Respondents were willing to pay about AUD 11 annually into a conservation fund (median value), including those who would pay nothing. Highest values were offered by young or middle aged men, and those with knowledge of birds and those with an emotional response to encountering an endangered bird. However, the prospect of a bird going extinct alarmed almost everybody, even most of those inclined to put the interests of people ahead of birds and those who resent the way threatened species sometimes hold up development. The results suggest that funding for threatened birds has widespread popular support among the Australian population. Conservatively they would be willing to pay about AUD 14 million per year, and realistically about AUD 70 million, which is substantially more than the AUD 10 million currently thought to be required to prevent Australian bird extinctions.

## Introduction

Wild birds have been the subject of economic valuation studies for decades [1], [2]. Direct economic values to humans [3] include being hunted for food or sport [4], [5], pest control [6] or as objects of tourism [7], [8]. However, birds also play less tangible roles that increase the well-being of those who encounter them [9]. Like many ecosystem services [10], [11], birds provide utility to humans in ways that are not traded in the market and so their value cannot be obtained from observations of market transactions. Put another way, many people would feel poorer should wild birds no longer enrich their daily lives but there is no market from which their presence can be purchased. Thus quantifying the economic value of wild birds, including values not directly related to use, requires non-market valuation techniques.

Stated preference methods, of which contingent valuation (CV) is one, allow for the estimation of non-market goods for which there is no corroborating market behaviour that would provide reliable measurements [12]. Stated preference methods have been used to value non-market environmental goods for more than 50 years [13], with few alternative methods [14]. The core of a survey-based CV is the creation of a hypothetical market where respondents are asked to state directly their willingness-to-pay (WTP) for the good in question based on information provided to them. Studies using CV have provided a range of meaningful quantitative estimates of the anthropocentric benefits derived from threatened species conservation [15]. Most WTP bird studies have evaluated single, often threatened, bird species [16]–[20], some considered a category of birds (e.g. migratory birds) [21], some investigated multiple threatened and non-threatened species of which one assessed category was birds [22]–[24] and some studies investigated particular qualities of birds (e.g. rarity) [25]. Some of the values identified in these studies have been substantial. A meta-analysis of 12 studies found a mean WTP of USD44 per threatened species per year [24]. More recently a CV study found an average WTP for the nationally threatened corncrake (*Crex crex*) of between €7 and €11 among Irish farmers [20].

In Australia 211 bird taxa have been assessed as threatened or Near Threatened using the IUCN Red List criteria [26]. Although investment in conservation of these species has already been substantial [27], preventing their extinction will still cost millions of dollars to pay for actions ranging from direct interventions for individual species through to opportunity costs incurred by retaining habitat that might otherwise be developed [28], [29]. Against this are the benefits from birds received by the Australian public who, for the most part, pay for conservation through their taxes. Comparing the costs and benefits (the value) of threatened bird conservation can lead to optimal conservation investment.

In this study we provide a monetary estimate of some of the benefits bird conservation would bring to Australians. Using the CV method, we compared the stated WTP across respondents from different socioeconomic backgrounds, with different attitudes and beliefs about birds and bird conservation, and with different levels of knowledge about birds. We know of no other study in which the diversity of perceptions about birds and the value of all threatened birds, rather than a narrow selection of species, has been estimated for a whole country.

## Methods

### Willingness-to-pay determinants

Many studies that have evaluated the value of threatened species and peoples' WTP for their conservation, have investigated and found variation in WTP across respondents. Most studies test for age, education, gender and income effects on the WTP, respondents' location (e.g. urban vs. rural) and the distance of the respondent to the species in question, respondents' knowledge of the species and their attitudes towards environmental issues and conservation more generally. The choice of factors thought likely to influence the WTP for threatened bird conservation in Australia, and so tested in this research, arose partly from literature review and partly from qualitative interviews prior to the survey. Apart from commonly used economic and demographic determinants such as income, gender and age, we were particularly interested in the impact of respondents' knowledge about bird identification and their attitudes towards threatened birds on their WTP. Table 1 outlines the variables that we test in this study and the expected impact on peoples' WTP for threatened bird conservation in Australia.

**Table 1:** Potential willingness-to-pay (WTP) determinants and their expected impact (positive [+]/negative [−]).

Determinant	Expected impact on WTP
Income	+
Being female	+
Age	−
Interest in birds in general	+
Attitudes towards threatened birds	
Aesthetic value	+
Humanistic value	+
Spiritual value	+
Scientific value	+
Experiential value	+
Existence value	+
Utilitarian value	−
Knowledge of birds, measured by peoples' self-rated ability to identify common birds	+

We expected that people with high incomes would be more likely to pay as well as to pay more for threatened bird conservation in Australia, as found for other threatened wildlife (e.g. for the conservation of black-faced spoonbills (*Platalea minor*) [19], for peregrine falcons (*Falco peregrinus*) and shortnose sturgeon (*Acipenser brevirostrum*) [17]). Age has been found to be a consistent predictor which is negatively related to WTP for environmental amenities in general [30] and, for example, for the recovery of the guillemot (*Uria aalge*) population in Spain [31] specifically. Being female is often positively associated with higher WTP for environmental amenities ([30]; and, for example, for biodiversity protection in Germany [32] and the conservation of Mediterranean monk seals (*Monachus monachus*) [33]), and so we hypothesise that women have a higher WTP for wild birds than men.

The attitudinal questions in our survey are based on the categorization of attitudes to wildlife developed by Kellert [34]. For each of Kellert's eight categories of wildlife value, we posed one statement question to each respondent. Research on environmental attitudes and WTP often find a positive relationship between the two [17], [23], [35].

Finally we integrated a knowledge variable as an indicator for peoples' WTP. This variable is a measure of respondents' self-rated ability to identify common birds. The possible answers were: cannot identify any, can identify some, can identify most and can identify all common birds. The expectation was that those people who have good or expert knowledge about birds, i.e. those who said they can identify most or all common birds, would be willing to pay more for the conservation of threatened species than those with less knowledge. Apart from examples in wildlife valuation where this was evident [16], [18], [36], this phenomenon was also found for the conservation of threatened livestock breeds [37].

### The sample

The survey was delivered online between 16^th^ and 21^st^ of February 2011. We opted for a cost-efficient online survey because other valuation studies have shown that WTP values do not vary significantly across different survey modes [38]–[40]. The survey was commissioned by a survey company, MyOpinions Pty Ltd, and respondents were paid AUD 3 on completion (at the time of the survey the AUD equalled the USD). The survey was voluntary and anonymous, and ethics approval was obtained from Charles Darwin University Human Research Ethics Committee (H11059). MyOpinions Pty Ltd is accredited to ISO 20252 and ISO 26362, adheres to the “research only” policy governed by industry bodies including the European Society for Opinion and Marketing Research, the Australian Market and Social Research Society and the Association of Market and Social Research Organisations and has an active panel of 300,000 verified respondents drawn from the general public (1.2% of Australian population) who registered (without having received any payment) with the company after recruitment via television, radio, newspaper, and online. Approximately half of the MyOpinions panel has been recruited from offline sources. The sample was selected using a quota random sampling whereby quotas were set to match the national population for gender, age and geographic location. The survey company randomly selected 5,800 members within these quotas and invited them to participate. Of these, 1,229 people agreed to undertake the survey before the topic was revealed. From these, 70 people dropped out before they started the survey. From the remaining 1,159 people, nearly 56% (645 people) completed the survey. The overall response rate of 11% (645/5,800) is consistent with other online surveys [41] where the invited sample tends to be very large to start with to ensure that all survey categories reach their quota quickly.

### Questionnaire

The questionnaire had four sections: 1) questions aiming to elicit respondents' attitudes towards birds and bird knowledge, 2) the CV question and a follow-up on the motivations for those not willing to pay, and 3) questions on socioeconomic characteristics (income, education, current employment situation and country of birth). For the CV we used a single-bounded dichotomous yes/no choice question on whether respondents were willing to pay for a stated amount (referred to as a bid) that varied between questionnaires [42], [43]. Additional to these bids we offered respondents the opportunity to state their WTP openly, which could be lower or higher than the bid. The number of bids and bid amounts were finalised after a pilot study with 30 respondents. The chosen bid amounts offered to respondents were (in AUD): 10, 20, 50, 100, 200. As a payment vehicle a contribution to a conservation fund was chosen and the payment was said to be yearly. The bid amounts were randomly rotated during survey distribution so that equal numbers of respondents within the sample group were shown each bid. The wording of the CV question was as follows:

*Of the money you might donate to any kind of cause (charitable, conservation etc.), would you be willing to pay the amount shown below per year into a conservation fund for threatened birds?(if you would not donate the amount shown below, please write your preferred amount into the box)*

*AUD [Bid] per year*

*Or, please specify your preferred amount AUD........*

All attitudinal questions were assessed using a Likert scale [44] format with five potential answers: ‘Strongly disagree’, ‘Disagree’, ‘Neither agree nor disagree’, ‘Strongly agree’, ‘Agree’. To rate respondents' overall interest in birds we asked them to rate their agreement to the statement ‘Seeing a new bird fills me with excitement’. We then asked respondents the extent to which they agreed with a set of value-related statements based on the following question: ‘Thinking about how you would feel if you knew you had seen an endangered bird, how much do you agree or disagree with these statements?’.

### Analysis of CV responses

A respondent's preference for the environmental good in question, and thereby the probability of accepting the bid over declining it, is based on the random utility framework, stating that utility is composed of two parts, a deterministic part which we can observe (v) and a random part (ε) that we cannot measure [45]. The utility (U) derived from environmental good (q) can be written as (y = income):
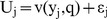
(1)Following Haab and McConnell [46] we assumed a linear utility function, so that the deterministic part of utility can be written as:
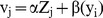
(2)Where Z is a range of respondents' socio-economic characteristics and y is income for respondent j. The probability that a respondent will accept the offered bid (BID) is then:
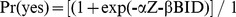
(3)Using a probit model we estimated the α's and β's and subsequently estimated both the mean and the median. Assuming a linear functional form both are given by
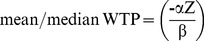
(4)and assuming an exponential functional form the mean is given by 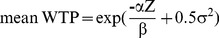
(5)and the median is given by 
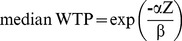
(6).To calculate the mean and median WTP values as well as the 95% confidence intervals (CI), we employed the Stata command ‘wtpcikr’ [47]. The CIs were calculated using the Krinsky-Robb approach [48], i.e., the standard errors are derived via simulation. For the simulation we used 10,000 draws. In addition to a bid-only model, which includes solely the constant and the bid parameters, we also estimated a model investigating whether the hypothesised WTP determinants are statistically significantly related to the responses toward the offered bids, i.e. accepting or declining them (Yes/No-response). This model is called the ‘Covariates model’.

## Results

### In general willingness-to-pay and protest responses

More than half the respondents (353; 55% of 645) did not want to contribute to a threatened bird fund in general. These people rejected the bid and also did not state an alternative amount that they might be willing to pay. A follow-up question after the CV asking respondents why they did not want to pay (Table 2) was used to separate those people who had zero value for threatened birds (valid ‘no’ responses) and those who opposed the CV question even though they might value threatened birds (protest responses). Most of the non-contributors said that they could not afford to pay (37%) or that they already donate money to another cause (32%). Some (11%) would not donate to any fund in general, while a few said that their taxes already support the protection of threatened birds (6%) or that they support bird conservation in other ways already (6%).

**Table 2:** Stated reasons for not contributing to a threatened bird conservation fund (in %).

Reason	N	% of those not contributing (N = 353)	% of whole sample (N = 645)	Response type
I already donate to a bird conservation fund	1	<1	<1	True zero value
I already donate to another cause	112	32	17	True zero value
I cannot afford to donate any money to a bird conservation fund	131	37	20	True zero value
I support bird conservation in other ways	20	6	3	True zero value
I would not donate to any fund like this in general	39	11	6	Protest
My taxes already support protection of endangered birds	21	6	3	Protest
No answer	21	6	3	True zero value
Other	8	2	1	True zero value

Respondents who have been identified as protesting against the payment vehicle are usually deleted from the sample [49]. However if those who are categorised as protesters actually have a WTP, then assuming a zero WTP for them would underestimate the economic value of the good in question [50]. On the other hand, the economic value could be overestimated if respondents categorised as protesters but having a zero WTP are ascribed some average value. Following Jakobsson and Dragun [51], we treated positive responses to two of the reasons as protest responses and deleted them from the data set. These were the 39 respondents who would not donate to any fund in general and 21 respondents who believed that their taxes already pay for the protection of threatened birds. This reduced the data set from 645 to 585 respondents. A further 17 responses could not be used because people did not answer most of the questionnaire properly. The final dataset contained 568 responses.

### Sample characteristics

With 61% of the 568 respondents being female, the sample constitutes a slight gender bias. Forty percent of the respondents had an income of up to AUD 40,000 per year, 27% of AUD 41,000-80,000 and 7%>AUD 80,000; the remaining 26% of respondents did not reveal their income category. In accordance with the predetermined sample request, respondents were distributed relatively evenly across all age categories (18–24∶10%, 25–34∶13%, 35–44∶17%, 45–54: 21%, 55–64∶17%, 65+:22%). Also by request, the geographical distribution of respondents matched the demographic variation among Australian states (New South Wales 31%, Victoria 25%, Queensland 20%, Western Australia 10%, South Australia 7%, Tasmania 4%, the Australian Capital Territory 2% and the Northern Territory 1%).

### Attitudes towards threatened birds and their conservation

The highest Likert sample means, over four, were found for the three statements ‘I would regret that humans had caused the bird to become endangered’, ‘I think there's a moral obligation to protect the bird’ and ‘I would feel upset if the bird became extinct’ (Table 3), indicating a strong dislike of endangered birds becoming extinct across the whole sample, including many who had a zero WTP. Of the 13% who actively rejected payments, 35% still agreed or strongly agreed that they would both ‘not like to see extinction’ and ‘feel a moral obligation to protect the bird’ with 43% regretting that human activities were making it threatened.

**Table 3:** Responses (in %) to a series of statement questions asking: ‘Thinking about how you would feel if you knew you had seen an endangered bird, how much do you agree or disagree with these statements?’

Statement	Strongly disagree (1)	Disagree (2)	Neither Agree nor Disagree (3)	Agree (4)	Strongly agree (5)	Sample Mean
Seeing a new bird fills me with excitement	4	16	32	33	15	3.4
I want to learn more about the bird	1	7	32	48	12	3.6
I want to add it to my bird watching list	14	24	40	18	4	2.8
I would regret that humans had caused the bird to become endangered	<1	2	12	47	39	4.2
I think there's a moral obligation to protect the bird	<1	1	17	49	33	4.1
I feel it's a nuisance when an endangered bird stops development	34	27	23	11	5	2.3
I think the bird has a right to live only if it's beautiful or unusual	45	28	13	8	6	2.0
I feel the needs of people come before those of endangered birds	26	32	32	8	2	2.3
I think government is responsible for the bird's survival, not me	17	38	35	8	2	2.4
I would feel upset if the bird became extinct	2	3	15	47	33	4.1
I would feel privileged or spiritually uplifted	1	4	31	43	21	3.8

To reduce the individual items to underlying latent factors (Table 4) we used factor analysis. Factor analysis is a family of approaches that aim to reduce a number of observed, correlated variables, such as responses to attitudinal questions, by describing linear combinations of the variables that contain most of the information. This information can subsequently be used to reduce the set of variables to a lower number of unobserved latent variables, also called factors [52], [53]. Prior to the factor analysis we calculated the Kaiser-Meyer-Olkin (KMO) Measure of Sampling Adequacy. With a value of 0.825 it indicates that the data are suitable for a factor analysis. Subsequently, the Varimax rotated results singled out one factor with an Eigenvalue of 2.2 which explains 63.6% of the variance. The Eigenvalue of a factor measures the variance in all the variables which is accounted for by this particular factor. The higher the Eigenvalue the more the factor contributes to the explanation of the variances in the variables [53]. Among the three remaining factors, only one had an Eigenvalue above one. However this value was only slightly above one (1.13) so we did not use it in the subsequent analysis. To calculate an attitudinal factor score for incorporation into the bid function, we used the items with factor loadings above 0.4. We called this score ‘Avicentric’. The higher this score, the more positive the attitude of a respondent towards threatened birds and their protection. The score had a mean value of 16.6 and ranged from a minimum value of 8 to a maximum value of 21.

**Table 4:** Results of factor analysis extracting four common factors explaining the correlations amongst responses to Likert-type statement questions.

Statement question	Factor 1	Factor 2	Factor 3	Factor 4
I think there's a moral obligation to protect the bird	0.75	−0.16	0.19	−0.02
I would regret that humans had caused the bird to become endangered	0.72	−0.17	0.14	−0.04
I would feel upset if the bird became extinct	0.58	−0.08	0.22	0.19
I would feel privileged or spiritually uplifted	0.54	−0.09	0.30	0.19
I want to learn more about the bird	0.47	−0.07	0.58	0.04
I might tick the bird off my bird watching list	0.25	0.13	0.57	−0.01
I think government is responsible for the bird's survival, not me	0.15	−0.43	0.14	0.05
I think the bird has a right to live only if it's beautiful or unusual	−0.14	0.59	0.05	0.01
I feel it's a nuisance when an endangered bird stops development	−0.19	0.55	0.05	−0.05
I feel the needs of people come before those of endangered birds	−0.34	0.44	−0.06	−0.03
Eigenvalue	2.19	1.13	0.87	0.08

Note: Responses to the first five statements with a loading higher than 0.4 were grouped into one variable which we called ‘Avicentric’.

### Responses to offered bids

In total, respondents accepted the offered bid in 25% of the CV questions while they rejected it in 75% of the questions (Table 5). The percentage of respondents accepting the bid diminished as its cost increased. Almost half of the respondents (45%) accepted the lowest bid offered (AUD 10) while only four respondents (3%) answered ‘yes’ to the highest bid of AUD 200.

**Table 5:** Distribution of responses to the WTP bids (in %); N = number of respondents offered the bid.

	Bid (in AUD)					
Response	10	25	50	100	200	Total
Rejected bid	55	57	78	87	97	75
Accepted bid	45	43	22	13	3	25
N	101	111	138	100	118	568

From those 428 respondents who did not accept the offered bid, 133 (31%) suggested a maximum WTP that was lower than offered in the CV question. A few respondents (17) had a higher maximum WTP than indicated by the bid they had accepted. The mean annual WTP of the former group was AUD 26.40. The respondents who professed a WTP that was higher than the bid had a mean annual WTP of AUD 115.30. The mean of the bids offered to those respondents was AUD 15 and thus was in the lower range of the offered bid vector.

### Bid function estimation

Various model specifications were tested incorporating different WTP determinants. The bid values were log-transformed. Based on the log-likelihood values, information criteria (AIC and BIC) and the pseudo R^2^ measure [54] we opted for the model presented in Table 6. This model showed the best performance among the model specifications tested. Overall, with a pseudo R^2^ of 0.20, the model performs well [54]. As expected, the bid amount was negative and statistically significant at the 1% level (Table 6). The negative sign denotes that the higher the bid amount the respondent was asked to donate, the lower the probability that the respondent would accept it. The extent to which people were excited about seeing a new bird and had positive attitudes towards bird conservation (Factor 1: ‘Avicentric’) significantly and positively affected the likelihood of accepting the bid. The higher the respondents' score for this attitudinal variable, the higher was their WTP. These positive impacts on peoples' WTP agreed with our expectations (see Table 1). The fact that people who could not identify any common birds were less likely to accept the offered bid was also consistent with our expectation, because people with poor knowledge of birds probably value them less than those who have made the effort to learn to identify most or all common birds. However, some determinants did not have the positive impact we had expected, e.g. income and younger age groups were insignificant. The only significant age group was ‘older than 65’, which had a negative effect on WTP. The gender effect was the opposite of what we expected with male respondents being more likely to accept the bids.

**Table 6:** Bid-only and covariates model (probit), depended variable = Yes/No response to offered bid.

	Bid-only model			Covariates model		
Variable	Coef.	SE+	p-value	Coef.	SE+	p-value
Constant	1.296	0.06	0.001	0.54	0.58	0.356
Bid (log)	−0.534	0.24	0.001	−0.60***	0.07	0.001
Age 25–34				−0.17	0.27	0.527
Age 35–44				−0.34	0.26	0.193
Age 45–54				−0.34	0.25	0.170
Age 55–65				−0.32	0.26	0.215
Age>65				−0.50**	0.25	0.049
Female				−0.27**	0.14	0.049
Medium income				0.14	0.15	0.366
High income				0.10	0.26	0.700
Can identify some common birds				0.11	0.18	0.539
Cannot identify any common birds				−0.41*	0.22	0.0610
Attitudinal score ‘Avicentric’				0.08**	0.03	0.0110
Excited to see birds				0.37**	0.15	0.0140
Log-likelihood _null_			−317.19		−317.19	
Log-likelihood _model_			−278.25		−254.10	
Pseudo R^2^			0.12		0.20	
AIC			560.50		539.67	
BIC			569.18		604.81	
Observations			568		568	

+ SE = Standard Error.

*** = 1% significance level; ** = 5% significance level; * = 10% significance level.

### Willingness-to-pay estimation and aggregation of estimates

The median WTP estimates for threatened birds in Australia were computed as between AUD 11.30 and AUD 11.55, for the bid-only model and the model including covariates, respectively (Table 7). These figures were aggregated for the population of working adults (rounded about 11 million [55]). The most conservative estimate (AUD 13.7–14.0 million) assumed that all those who did not accept the invitation to participate in the survey (89% of those requested) had a zero WTP, even though the subject of the questionnaire was not revealed in the invitation. A more realistic, but still conservative, estimate (AUD 69.6–71.1 million) assumed that all of those who failed to complete the questionnaire after accepting the invitation to participate had a zero WTP (44%; Table 7).

**Table 7:** WTP estimates (in AUD) for Australian threatened bird conservation and aggregation of these estimates.

Variable	Bid-only model	Covariates model
Mean WTP	65.10 [42.27–166.00]	46.61 [33.46–90.35]
[95% CI]		
Median WTP	11.30 [7.16–15.21]	11.55 [7.70–15.23]
[95% CI]		
Aggregation based on median WTP		
Conservative scenario: 11% of adult Australians+ would pay the average median WTP1)	13,673,000	13,975,500
Realistic scenario: 56% of adult Australians would pay the average median WTP2)	69,608,000	71,148,000
		

+ There are about 11 million adult Australians (rounded; [55]).

1) This assumes that all of those people who did not respond to the survey when invited by the survey company (89%) have a zero WTP for threated bird conservation in Australia.

2) This assumes that all of the 44% who did not complete the survey have a zero WTP and with the other 56% having a WTP corresponding to the sample.

## Discussion

Based on data collected in the 1990s, about AUD 5 million per year for the next 80 years could reduce Australian bird extinctions to almost zero and reduce the total number of threatened species by 15% [28]. Even assuming this figure has doubled to about AUD 10 million a year [56], this is still less than one dollar a year for the 11 million Australian adults of working age. Respondents to our survey were willing to pay over ten times that amount, around AUD 11 per year (median value), for threatened bird conservation in Australia, even including the non-contributors. This suggests that, even if we assume that all 89% of those who did not respond to the invitation to participate in the survey would be unwilling to pay, an adequate allocation of public funds to threatened bird conservation would be consistent with the benefits gained by the Australian public.

That more than half of the sample were not prepared to pay may also be deceptive. Three percent said they already supported bird conservation in other ways. Three quarters of the 20% unwilling to pay because they could not afford to (see Table 2), nevertheless agreed that humans have a moral obligation to conserve birds. Of the protest votes, 3% felt that government already covered their responsibility towards birds and just 6% actively rejected payment into a conservation fund. Curiously nearly half of the protest group (47%) said they would still pay for threatened birds in general and the majority (83%) would be upset if a bird went extinct. This survey therefore demonstrates that there is substantial support for the conservation of threatened birds across society. It also shows that threatened birds are valued as a group, not just particular species with which people might have a strong affinity.

This strong desire among respondents for wild birds not to go extinct is perhaps surprising given the relatively low rate of membership of bird clubs in Australia. BirdLife Australia has about 25,000 members, supporters and volunteers and around 12,000 Australians participate in BirdLife Australia's Birds in Backyards citizen science program [57], [58] (0.3% of the Australian population) with others interested in birds belonging to natural history and avicultural societies (which collectively are likely to have many more members than BirdLife Australia). This is far lower than, for example, the United Kingdom where the Royal Society for the Protection of Birds has over a million members (1.7% of the population, about one in every 60 people of all ages). In the survey 2.5% were or had been a member of a bird club of any type, although 19% said they sometimes went birdwatching. International comparisons of WTP for threatened birds could provide meaningful comparisons of conservation culture, although the comparison would also need to take account of national differences in the probability of joining a society of any type.

Given that this WTP was elicited from people who had not been primed for the questionnaire, one might expect that it would have been higher had people had greater knowledge than provided in the survey introduction. The result that the stated WTP in this study was higher among those more knowledgeable about birds, and that increasing a person's knowledge about a species increases their stated WTP [18], suggests that there is considerable extra support that could be generated if more people knew about the species that could be lost. While stated and actual WTP differ, there was, no suggestion that interest in conservation peaked among those moderately well-informed (unlike [59]). Similarly there was no suggestion to confirm our hypothesis, in line with other similar studies [33], that women are more likely to support conservation than men, even though men tend to relate differently to birds than women [60]. There was, however, an effect of age, with respondents over 65 being less likely to pay, which is in line with the attitudes of young people towards taking environmental responsibility [61]. This confirmed our hypothesis of a negative relationship between age and WTP [30] with older people (beyond work force age) potentially not able to contribute as much money as younger people [31]. While people in Australia, as in some other wealthy countries like the USA and Japan, may be less directly involved with wildlife than they were historically [62], there still appears to be a strong wish among young people to prevent species extinctions, which is consistent with a substantial interest in retaining wildlife even without seeing it [18], [63].

The attitudinal values of those willing to pay for bird conservation also affected their WTP. Thus, as with Spash [23], who found a positive correlation between respondents who held the view that endangered birds have the right to protection and their WTP, there was a positive correlation between WTP and being ‘avicentric’. The results are also in line with those presented by Kotchen and Reiling [17] who found that a positive correlation between environmental concern, measured by the NEP (New Ecological Paradigm) scale, and WTP for species protection. However not all results were as expected: 55% of respondents who considered people more important than birds and 42% of those who resented threatened birds impeding development were still willing to contribute to retain them in general. Likewise, 58% of those thinking that a threatened bird has a right to live only if it is beautiful or unusual would still, in principle, be prepared to pay into a conservation fund. Thus, even among those who put the interests of people first, there was a willingness to contribute to bird conservation at sites where there were fewer trade-offs, and an unwillingness to condone extinction. Thus these apparently negative attitudes do not necessarily exclude people from wanting to conserve threatened birds. Generally, however, people expressing a strong emotional response to birds were willing to pay more than those interested simply in seeing them.

Caution is always required with WTP estimates. In this case the major caution is that, while the survey with the dichotomous choice format had an incentive compatible question format, the payment vehicle is not among those that support consequentiality and thus truth telling [43]. Hence we opted for a voluntary payment into a conservation fund as the payment vehicle, rather than a tax increase, which would be compulsory across the entire population. However, in Australia the link between tax and expenditure by government is nearly always indirect, and suggesting a tax increase may have confounded considerations of the value of birds with views about taxation increases in principle. We therefore felt that the link between a conservation fund and conservation action was more explicit and self-evidently voluntary. Also, while the choice of payment vehicle may have led to over-estimation of the WTP, the aggregation is based on the more conservative median WTP, which is lower than the mean. Moreover, some respondents stated that they were willing to pay higher amounts than they were requested to pay based on the bids and some respondents who rejected the offered bid were willing to pay lower amounts, which were on average higher than the estimated median WTP. Overall, we therefore think that the figures presented are sufficiently accurate as an estimate of the Australian population's valuation of threatened birds that they can be used in conservation policy decisions.

## Conclusions

There was strong support for the conservation of threatened birds among the Australian public as demonstrated by their willingness-to-pay for their conservation. Nearly half of the respondents said they were willing to pay into a bird conservation fund or did so already. Many of the remainder said they could not afford to pay rather than they would rather not do so. While support was strongest among those with a passion for birds and those who knew most about them, it was by no means confined to this sector of society. Even many of those who would favour development over birds would still be willing to pay to prevent extinctions. The study is notable for valuing a threatened fauna in its entirety rather than any specific bird. It also suggests that funding of threatened species conservation has broad backing from the Australian population.
